# Mechanical Characterization and Modeling of the Porcine Cerebral Meninges

**DOI:** 10.3389/fbioe.2020.00801

**Published:** 2020-08-31

**Authors:** Baptiste Pierrat, Louise Carroll, Florence Merle, David B. MacManus, Robert Gaul, Caitríona Lally, Michael D. Gilchrist, Aisling Ní Annaidh

**Affiliations:** ^1^School of Mechanical & Materials Engineering, University College Dublin, Dublin, Ireland; ^2^Mines Saint-Étienne, Centre CIS, Univ Lyon, Univ Jean Monnet, INSERM, U 1059 Sainbiose, Saint-Étienne, France; ^3^School of Mechanical & Manufacturing Engineering, Dublin City University, Dublin, Ireland; ^4^Trinity Centre for Biomedical Engineering, School of Engineering, Trinity College Dublin, Dublin, Ireland; ^5^School of Medicine and Medical Science, UCD Charles Institute of Dermatology, University College Dublin, Dublin, Ireland

**Keywords:** dura mater, mechanical properties, multiaxial testing, bulge inflation, SALS, meninges, traumatic brain injury

## Abstract

The cerebral meninges, made up of the *dura, arachnoid*, and *pia mater*, is a tri-layer membrane that surrounds the brain and the spinal cord and has an important function in protecting the brain from injury. Understanding its mechanical behavior is important to ensure the accuracy of finite element (FE) head model simulations which are commonly used in the study of traumatic brain injury (TBI). Mechanical characterization of freshly excised porcine *dura-arachnoid mater* (DAM) was achieved using uniaxial tensile testing and bulge inflation testing, highlighting the dependency of the identified parameters on the testing method. Experimental data was fit to the Ogden hyperelastic material model with best fit material parameters of μ = 450 ± 190 kPa and α = 16.55 ± 3.16 for uniaxial testing, and μ = 234 ± 193 kPa and α = 8.19 ± 3.29 for bulge inflation testing. The average ultimate tensile strength of the DAM was 6.91 ± 2.00 MPa (uniaxial), and the rupture stress at burst was 2.08 ± 0.41 MPa (inflation). A structural analysis using small angle light scattering (SALS) revealed that while local regions of highly aligned fibers exist, globally, there is no preferred orientation of fibers and the cerebral DAM can be considered to be structurally isotropic. This confirms the results of the uniaxial mechanical testing which found that there was no statistical difference between samples tested in the longitudinal and transversal direction (*p* = 0.13 for μ, *p* = 0.87 for α). A finite element simulation of a craniotomy procedure following brain swelling revealed that the mechanical properties of the meninges are important for predicting accurate stress and strain fields in the brain and meninges. Indeed, a simulation using a common linear elastic representation of the meninges was compared to the present material properties (Ogden model) and the intracranial pressure was found to differ by a factor of 3. The current study has provided researchers with primary experimental data on the mechanical behavior of the meninges which will further improve the accuracy of FE head models used in TBI.

## 1. Introduction

Traumatic brain injury (TBI), caused by a fierce acceleration or impact to the head, is a critical public health problem throughout the world (Peeters et al., 2015; MacManus et al., 2017a). In the U.S. alone, TBI accounts for 1.7 million injuries, 275,000 hospitalizations and 50,000 deaths annually (Faul et al., 2010). Its primary causes are road traffic accidents and falls (Peeters et al., 2015), while contact sports athletes are particularly susceptible to mild TBI (Gardner and Yaffe, 2015). Since the 2000s, both multibody dynamics models and finite element (FE) head models have been developed by researchers to better understand the response of the human head to dynamic loads (Gilchrist et al., 2001; Horgan and Gilchrist, 2003; King et al., 2006; Kleiven, 2006; Chafi et al., 2009; Li et al., 2011). These models have since provided insights into the fundamental mechanisms of TBI in road traffic accidents (Kleiven, 2007), accidental falls (Doorly and Gilchrist, 2006; Raul et al., 2006; Pascoletti et al., 2019), impact sports (Post et al., 2013), and ballistics impacts (Li et al., 2016). The accuracy of such models relies heavily on the accuracy of the constitutive data which underpins the tissue behavior. While there is ongoing interest in the mechanical characterization of brain tissue (Rashid et al., 2013), cranial bone (Motherway et al., 2013), and scalp tissue (Trotta et al., 2018a,b; Trotta and Ni Annaidh, 2019), the cerebral meninges and its importance in head impact biomechanics has been largely overlooked in recent years. The *dura mater* (DM), the outermost and most substantial of the tri-layer meninges, is one of the stiffest membranes in the human body (Van Noort et al., 1981a) and previous works strongly suggest that the DM helps protect the brain against damage (Van Noort et al., 1981a). A recent study by MacManus et al. (2017a) has shown that it can mitigate the dynamic response of the brain by reducing the shear stress and the peak intracranial pressure, when the scalp and skull is removed. The DM lines the inner surface of the skull and its main functions are to collect venous blood and to protect and support the brain and the spinal cord (Hamann et al., 1998). Researchers agree that the *lumbar* DM exhibits significant anisotropy (Runza et al., 1999), with the stiffness differing by up to 500%, however, the anisotropy of the *cerebral* DM remains unclear. Van Noort et al. (1981b), using polarized light and Hamann et al. (1998), using small angle light scattering (SALS) to analyse the collagen fiber architecture of human cerebral DM concluded that while there are regions of highly aligned collagen fibers, this alignment is only seen over short distances and the global structure of the cerebral DM can be considered as isotropic.

Currently, not all FE head models include a description of the meninges: of those that do (Horgan and Gilchrist, 2003; King et al., 2006; Kleiven, 2006; Chafi et al., 2009; Li et al., 2011), they often include a simplified linear response based on uniaxial tests performed by Galford and McElhaney (1970). There is a relative dearth of primary data on the mechanical properties of the meninges, and with the exception of Walsh et al. (2018); De Kegal et al. (2017) and MacManus et al. (2017a), all other studies have been conducted over 30 years ago (Galford and McElhaney, 1970; Van Noort et al., 1981a; McGarvey et al., 1984; Bylski et al., 1986). Of these previous investigations, there are large variations in results which could be attributed to the nature of the testing protocol, the source of the meninges samples, storage of samples and insufficient sample sizes. In addition, the main test method found in previous investigations is either uniaxial or biaxial tensile testing. There is only one other study which uses bulge inflation testing (Chauvet et al., 2010), a common experimental method used for similar membrane materials, however the purpose of that study was to compare the efficacy of different surgical suturing methods. Inflation testing offers a simple multiaxial test method for membrane materials (Avril and Evans, 2017), simply relating pressure to stresses and deflection to strains. In addition to uniaxial tension, this method has been chosen because it more closely replicates the *in vivo* loading condition of the meninges under intracranial pressure. Intracranial pressure (ICP) is the pressure of the cerebrospinal fluid and is an important variable in many medical conditions including stroke hydrocephalus or brain tumors (Syed et al., 2016).

In the present study, we address the dearth of primary experimental data on the cerebral meninges, and provide new constitutive data for inclusion in updated FE head models. This is achieved through uniaxial tension and inflation testing of fresh adolescent porcine DAM samples and through a structural analysis using SALS. We also seek to highlight the importance of the meninges in the mechanical response of FE head models under complex loading, e.g., craniotomy surgery following elevated ICP.

## 2. Materials and Methods

### 2.1. Sample Preparation

**Figure 1:**
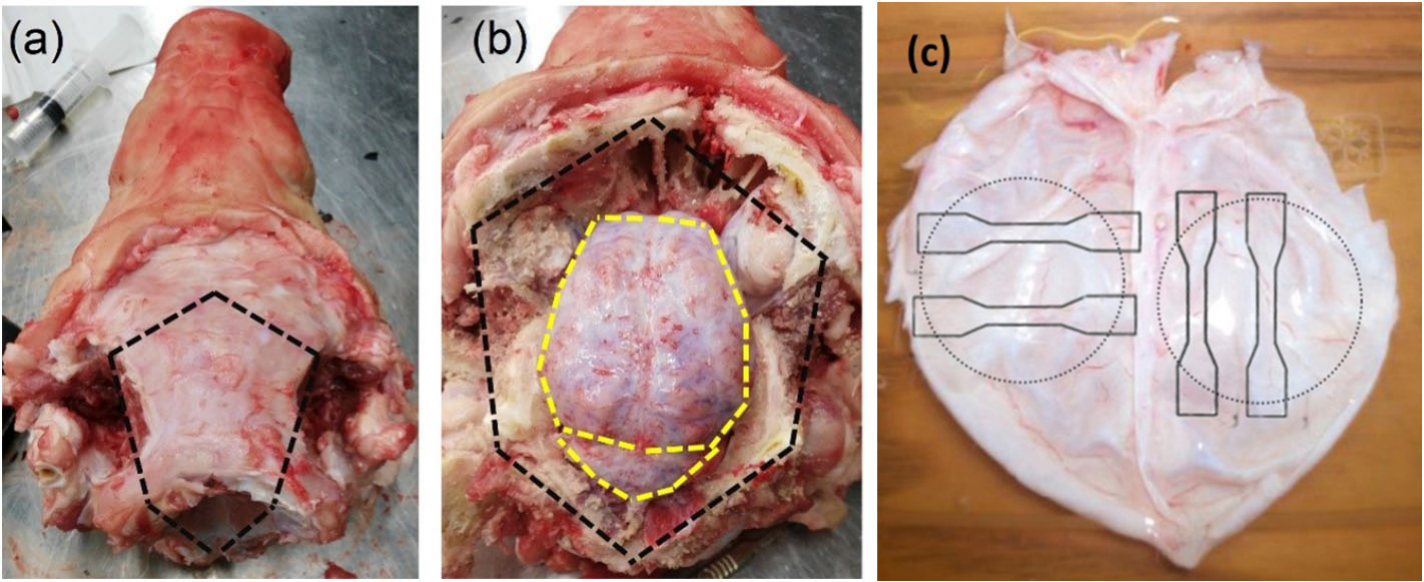
**(a)** Pig skull with scalp removed, skull to be removed by incising along black dashed lines. **(b)** Skull removed along black dashed lines, meninges to be removed by incising along yellow dashed lines. **(c)** Orientation and geometry of DM samples for uniaxial tensile tests (solid lines) and inflation tests (dotted lines) (MacManus et al., 2017a).

Post-mortem porcine specimens were collected from a local slaughterhouse and transported to the laboratory. The specimens consisted of six 22 week old (tensile tests) and five 23 week old (inflation tests) mixed sex pig heads. Previous studies have indicated that porcine tissue is a suitable model for both human brain and human meninges tissue (Frink et al., 2011; Mazgajczyk et al., 2012). To gain access to the cranial meninges and underlying brain tissue the scalp was removed from the specimens using a scalpel, exposing the cranial bone. Following the removal of the scalp, the cranial bone was excised using an oscillating saw. Incisions using the oscillating saw were made in a pentagonal shape around the top, sides, and back of the skull, as depicted in Figure 1a (dashed black line). Where possible, the incisions were made outside of the cranial cavity housing the brain to ensure the meninges and brain tissue were not damaged. Following removal of the skull and exposure of the meninges, the *dura mater* and *arachnoid mater* layers (DAM)^1^ of the meninges were removed using surgical scissors by incising along the yellow dashed line (shown in Figure 1b). The underlying tissue, e.g., cerebral cortex and pia mater, were left within the cranial cavity. The cerebral DAM was then tested immediately under ambient conditions (4 h after sacrifice) or was refrigerated in damp tissue for a maximum of 24 h before testing. As dehydration occurs within minutes, each sample was prepared and tested individually, while remaining samples were kept in saline solution to ensure that they did not dehydrate.

### 2.2. Tensile Testing

Samples were cut in a dogbone shape, of overall length 63.5 mm and width 3.2 mm. Sandpaper was glued to each end of the sample to reduce slipping in the grips. The gauge length and width of samples were measured optically from the camera, while the sample thickness was taken as an average of three measurements along the sample using Vernier calipers. Tensile testing was performed on a Hounsfield universal testing machine with a 1 kN load cell and a test speed of 50 mm/min (strain rate of 0.01 s^−1^). Overall, 25 samples from six pigs were tested until failure. The majority of samples tested (*N* = 17) were in the sagittal (longitudinal) orientation, however to investigate the possible anisotropy of the DAM, a number of samples (*N* = 8) in the coronal (transverse) orientation were also tested. Three DAMs were tested so that both longitudinal (*N* = 6) and transverse (*N* = 8) samples from the same DAM could be compared (as shown in Figure 1c), thereby eliminating inter-subject variability as a factor.

#### 2.2.1. Ogden Hyperelastic Model

The incompressible deviatoric strain energy density function for the Ogden model, *w*_*o*_, is given by Equation (1).

(1) $$\begin{array}{r} w_{o}=\frac{\mu }{\alpha }\left(\lambda_{1}^{\alpha }+\lambda_{2}^{\alpha }+\lambda_{3}^{\alpha }\right) \end{array}$$

where μ is the first Ogden parameter and α is the second Ogden parameter (Ogden, 1997). For an incompressible material in uniaxial tension, λ_1_=λ and λ_2_=λ_3_=$\frac{1}{\sqrt{\lambda }}$. Therefore, *w*_*o*_ becomes:

(2) $$\begin{array}{r} w_{o}=\frac{\mu }{\alpha }\left(\lambda^{\alpha }+2\lambda^{-\frac{\alpha }{2}}-3\right) \end{array}$$

The engineering (first Piola-Kirchhoff) stress in the tensile direction, *P* is given as:

(3) $$\begin{array}{r} P=\frac{dw_{o}}{d\lambda }=\mu \left(\lambda^{\alpha -1}-\lambda^{-\frac{\alpha }{2}-1}\right) \end{array}$$

The Cauchy stress in the tensile direction, σ is derived as

(4) $$\begin{array}{r} \sigma =\lambda P=\mu \left(\lambda^{\alpha }-\lambda^{-\frac{\alpha }{2}}\right) \end{array}$$

The parameters μ and α can be related to the initial shear modulus, μ_*s*_, by Equation (5):

(5) $$\begin{array}{r} 2\mu_{s}=\mu \alpha \end{array}$$

Finally the Young's modulus, *E*, can be related to the shear modulus with the following equation for an isotropic, incompressible material:

(6) $$\begin{array}{r} E=3\mu_{s} \end{array}$$

Similarly, the incompressible deviatoric strain energy density function for the NeoHookean model, *w*_*nh*_, is given by Equation (7).

(7) $$\begin{array}{r} w_{nh}=C_{1}\left(\lambda^{2}+2\lambda^{-1}-3\right) \end{array}$$

where *C*_1_ is the NeoHookean material parameter given by:

(8) $$\begin{array}{r} C_{1}=\frac{\mu_{s}}{2} \end{array}$$

Best fit Ogden and NeoHookean model parameters for each experimental stress-strain curve were obtained using the non-linear least squares function, lsqnonlin, in MATLAB.

### 2.3. Bulge Inflation Testing

**Figure 2:**
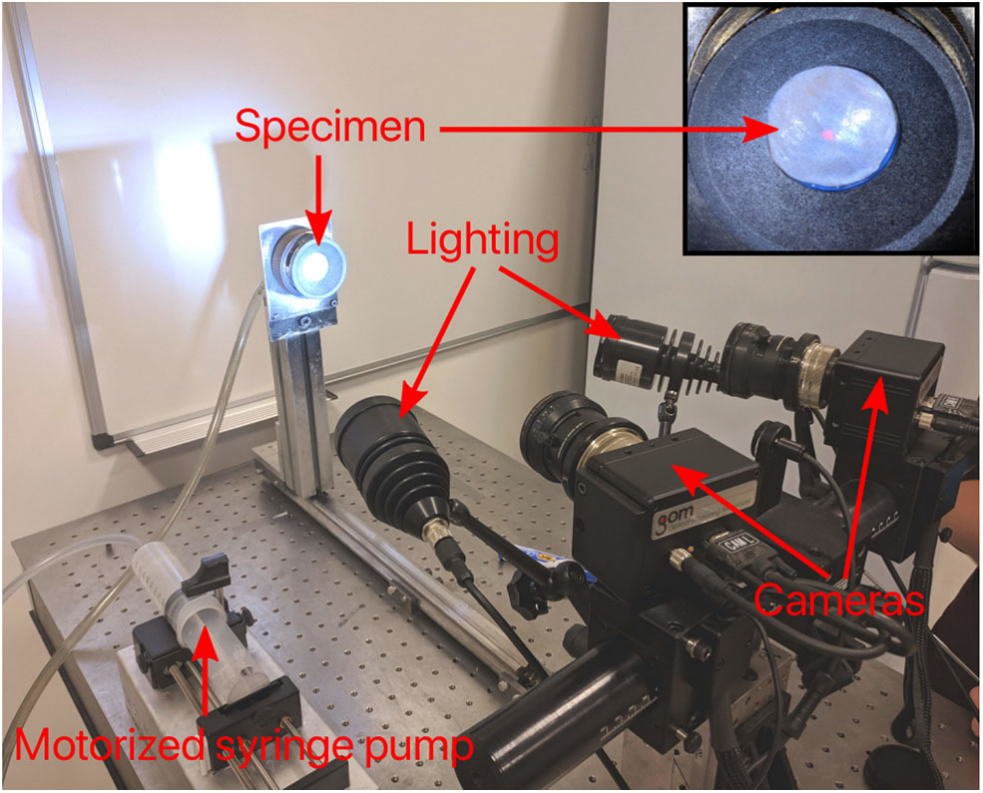
Inflation setup with stereo acquisition system. Main components include a motorized syringe pump to provide pressurized water to the specimen, which is clamped on its edges to seal the chamber; a pressure transducer to monitor the pressure and; two cameras (GOM, 5M LT) with a lighting system to acquire images for stereo vision.

A customized device was used to inflate the DAM and observe its deformation using stereo digital image correlation. The major components of the device include a motorized syringe pump containing water, a pressure transducer and a 30 mm diameter circular specimen holder (Figure 2). A stereo-camera bench was also used to capture a pair of images every second which was synchronized with the pressure transducer.

Five DAMs were cut into circular samples of ~40 mm diameter. All tests were performed until rupture or irreversible leakage. A constant volume rate was used to control the pump so that the pressure increased at a mean rate of 2.9 kPa/s.

**Figure 3:**
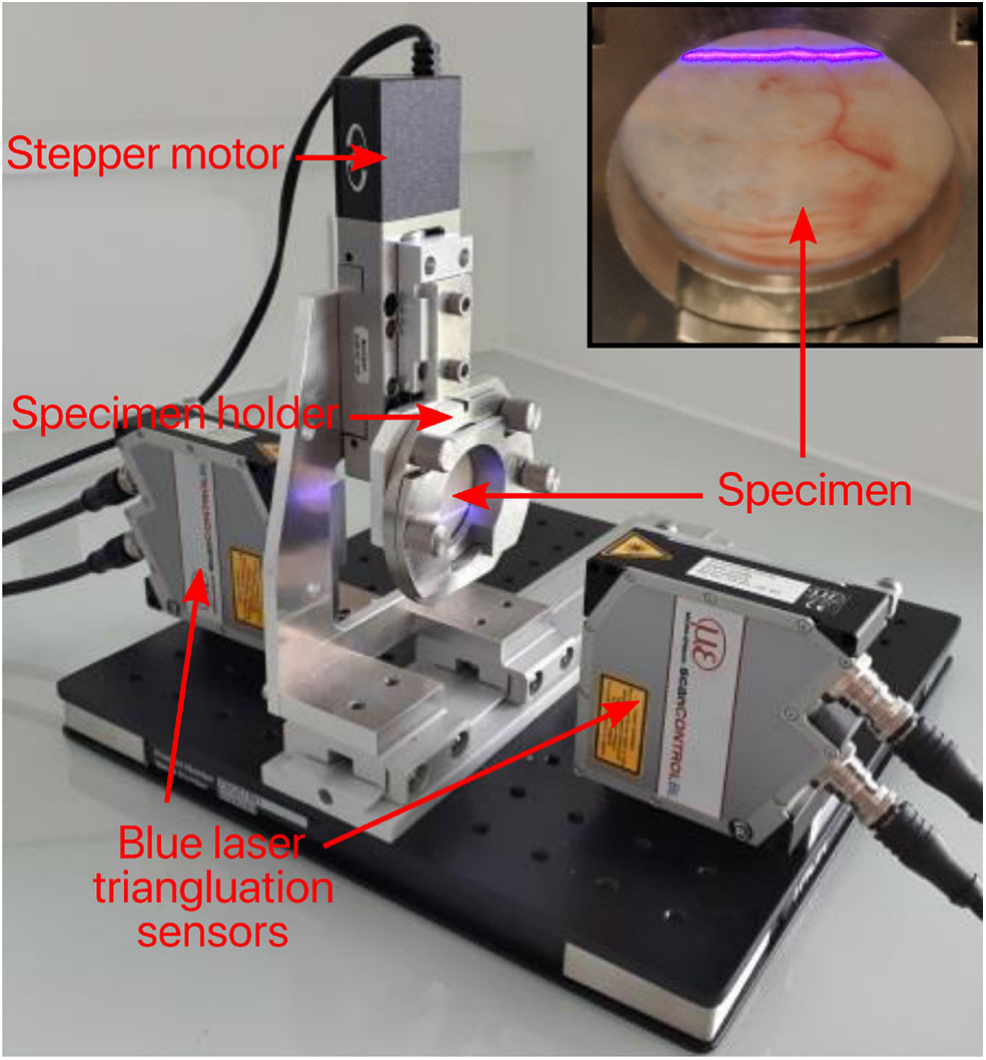
Image of thickness measurement device. Main components include two blue laser triangulation sensors which generate two distance maps to characterize the local thickness.

As bulge inflation tests are known to be particularly sensitive to specimen thickness (Rivlin and Saunders, 1951), great care was taken in its measurement. Prior to testing, the mean thickness of each DAM specimen was measured using a custom-made device (Figure 3) based on two blue laser triangulation sensors (Cavinato et al., 2019). Each laser is located on either side of the membrane where they both perform a scan of their respective surface to obtain two distance maps. The differential distance map then characterizes the local thickness, which is then averaged for the whole area of interest on the sample. Finally, a fine speckle pattern was sprayed on the samples with a graphite powder paint. During sample preparation and testing, great care was taken to keep the specimen hydrated.

#### 2.3.1. Initial Shape Measurement

**Figure 4:**
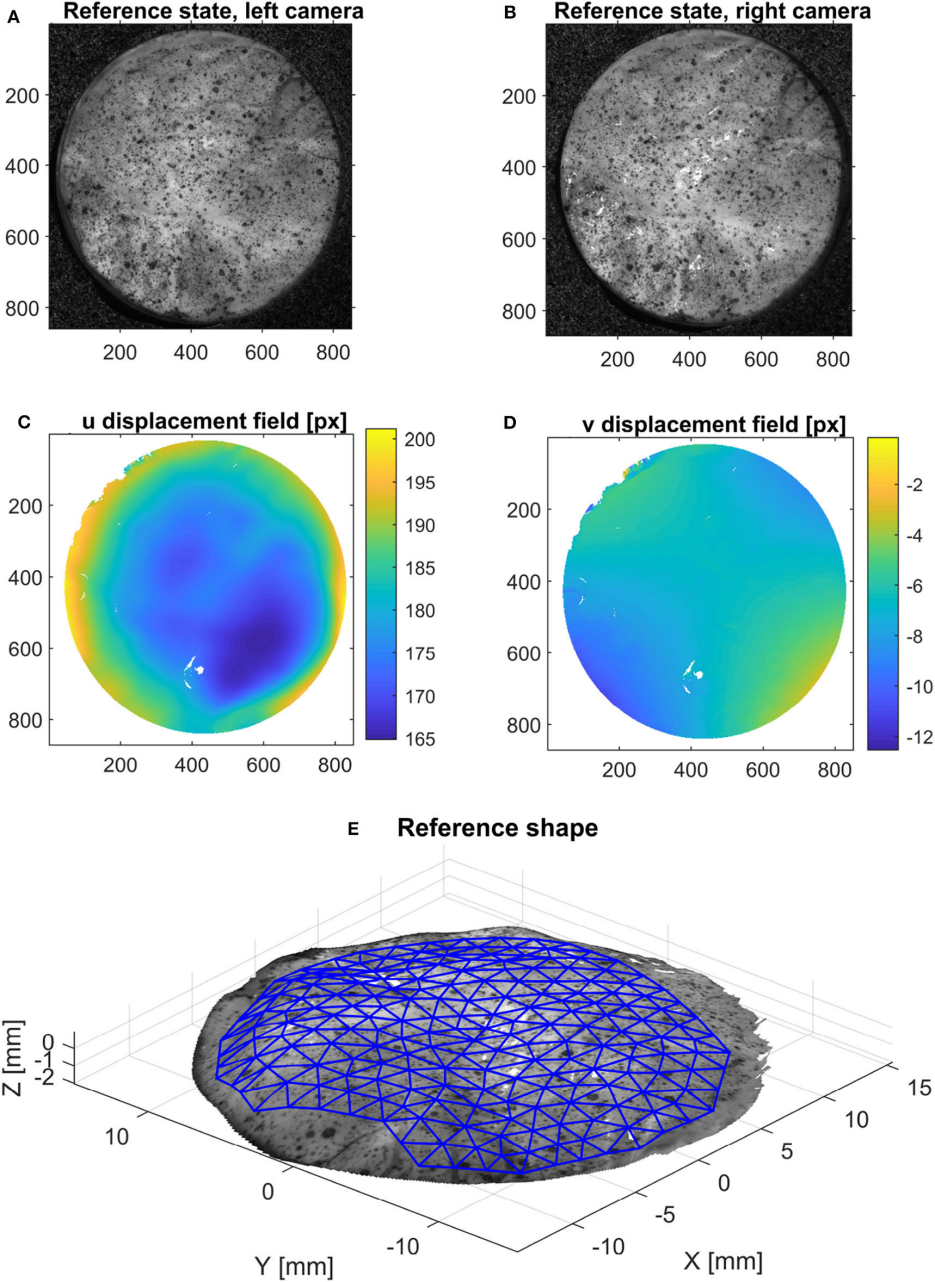
Method used to acquire the initial configuration using stereo-DIC: using the reference configuration [left **(A)** and right **(B)** images], the horizontal **(C)** and vertical **(D)** displacement fields between these pictures are calculated using 2D DIC. Then, triangulation provides the 3D surface of the object **(E)**, on which an area of interest is meshed for further processing.

The intrinsic and extrinsic camera parameters were determined using a chessboard pattern with the Stereocalibration tool in Matlab. Based on this calibration, it is possible to triangulate the 3D position of a point based on its pixel coordinates in the images from both cameras. In order to find the point correspondences between the two images at the reference state (horizontal and vertical displacement in pixels), an open-source digital image correlation software [NCorr (Blaber et al., 2015)] was used, as shown in Figure 4. This step yields a dense 3D point cloud (~400,000 points for the area of interest) of the surface of the specimen in the initial, undeformed configuration. In order to follow the deformation of the surface in the following states, the shape is interpolated on a coarser triangular mesh, as shown in Figure 4. A quadratic LOWESS interpolation robust to outliers was used for this purpose.

#### 2.3.2. Deformed Shape Measurement

**Figure 5:**
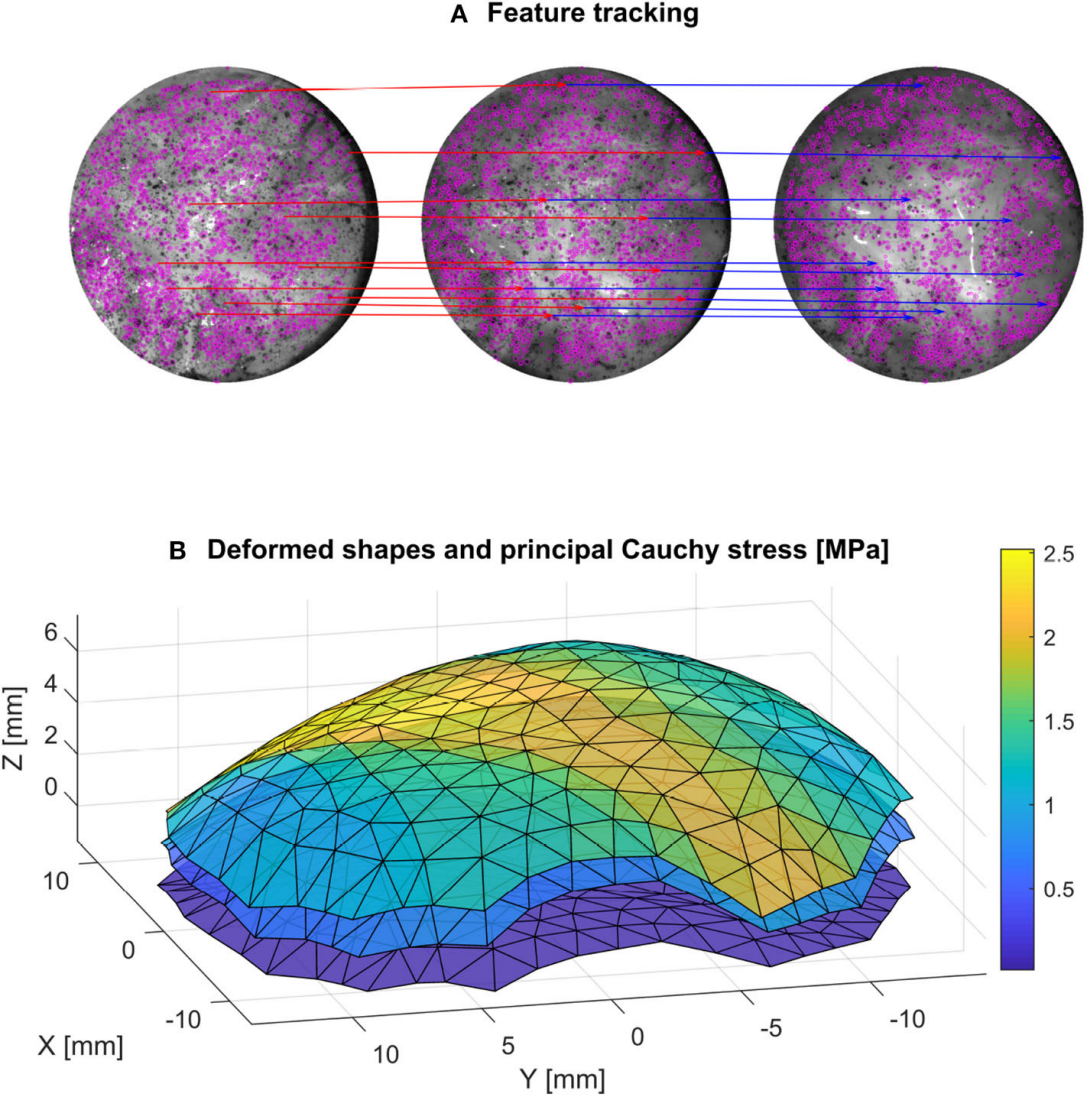
A feature tracking algorithm **(A)** is used (the tracking of 10 points for an initial, intermediate and final configuration is shown for better readability) to get the deformed configuration of the mesh at different stages **(B)**. The calculated principal Cauchy stress is also represented on the mesh for these three stages.

A fundamental experimental challenge noticed during the inflation tests was the slight porosity of the DAM. This lead to water droplets slowly leaking out of the surface and removing the speckle pattern in some locations, as seen in Figure 5. Instead of using a waterproof liner, which would have affected the global mechanical response of the sample, it was decided to base the processing of the deformed images on the remaining pattern that was not altered during the test. For this purpose, instead of a dense DIC technique, a sparse feature-tracking algorithm was used to track the speckle points that remained intact during the entire test. Such techniques have worse accuracy than DIC in terms of sub-pixel displacement, but are more robust to large displacements and by nature only track areas with high information. In this case, the TrackMate plugin (Tinevez et al., 2017) in imageJ was used to detect the features using a difference of gradient algorithm, and then track the displacement of the speckle features on the left and right cameras (Figure 5). Only the features that were tracked for the whole set of images were retained. Obvious outliers were removed based on the mean velocity of the tracked features. The nodal displacement of the mesh was obtained by interpolation of the sparse displacement field of the tracked features using again a quadratic LOWESS interpolation.

#### 2.3.3. Local Strain Measurement

The local strain is calculated based on the mesh configuration at each frame, using the same method employed by Volino et al. (2009). Briefly, the in-plane deformation gradient of each triangular element is computed directly from the nodal positions. Two initially perpendicular unit in-plane vectors *U* and *V* are defined in the initial configuration for each element, such as *U* is the projection of the medio-lateral direction on the element and *V* is the complementary vector representing the antero-posterior direction. These vectors are then updated based on the nodal position in the deformed configuration. The in-plane element deformation gradient is then:

(9) $$\begin{array}{r} \text{F}=\left[U\;V\right] \end{array}$$

The true (logarithmic) in-plane strain tensor is:

(10) $$\begin{array}{r} \text{E}=\frac{1}{2}\log\left({\text{F}}^{T}\text{F}\right) \end{array}$$

The strain in the third direction, i.e., the thickness variation, was computed based on initial and current thickness as described in the following section. These quantities were calculated for each element at every frame.

#### 2.3.4. Local Stress Measurement

Under a pure membrane assumption, it is possible to calculate the local Cauchy stress at each element knowing the pressure and the membrane shape. This procedure is described in Romo et al. (2014), Cavinato et al. (2019), and summarized below.

The mesh is composed of *n*_*elements*_ elements and *n*_*nodes*_ nodes. Based on force equilibrium at each element (the normal force due to the pressure equilibrates the local membrane forces of the three nodes), it is possible to write a set of 3 × *n*_*elements*_ equations relating the three in-plane stress components expressed at each node (so that the total number of unknowns is 3 × *n*_*nodes*_). Besides, additional equations can be added at the edges of the membrane so that the edge traction vector is normal to the edge direction (no shear) and in the plane of the membrane (no moment). In these equations, the thickness needs to be known. The initial thickness *h*_0_ is given by the local thickness measurements averaged over the element and the current thickness *h* is calculated based on the assumption of incompressibility:

(11) $$\begin{array}{r} h=\frac{h_{0}}{F_{11}F_{22}-F_{21}F_{12}} \end{array}$$

This overdetermined system of equations is solved using least-square matrix inversion for the nodal stresses, which are then distributed over the elements. The in-plane Cauchy stress tensor was calculated for each element at every frame with this method, as seen in Figure 5.

#### 2.3.5. Local Failure Stress Measurement

**Figure 6:**
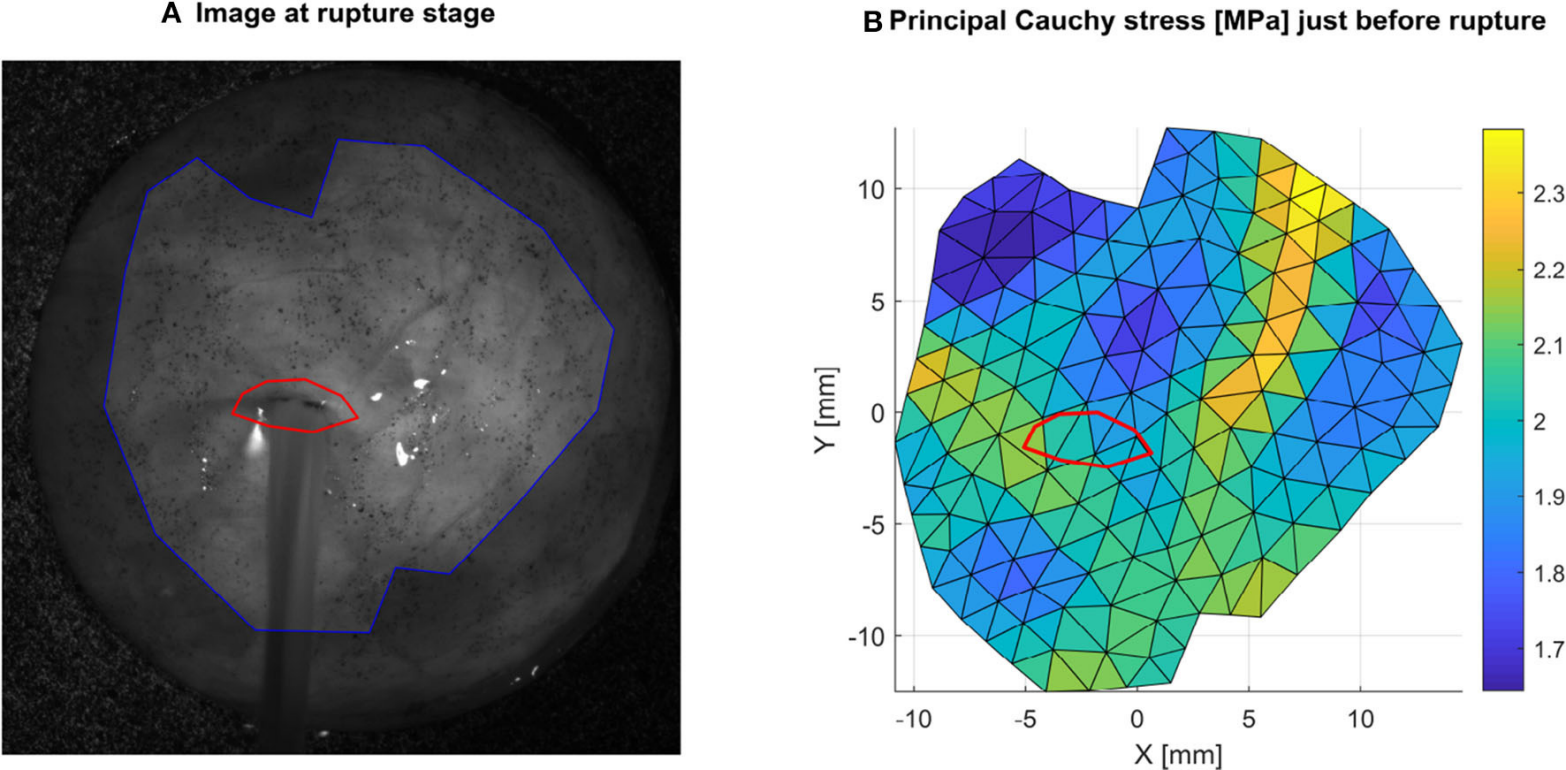
Local rupture stress is obtained by selecting the ruptured area **(A)** in red, and averaging the local principal Cauchy stress in this area **(B)** in the frame preceding rupture. Here an example is shown for sample 1R where the average value is 2.0 MPa.

On the first frame showing visible damage, such as water bursting from the sample, the damaged area was manually selected (Figure 6). Then, the local principal Cauchy stress of the previous frame just before failure was averaged in this area to characterize the local failure stress. Note that this process was performed only for tests showing failure in an area where shape reconstruction was successful and away from the clamped areas.

#### 2.3.6. Global Ogden Model Identification

Based on the local element stress and strain response throughout the inflation test, it would be possible to identify the parameters of a constitutive law for each element. However, due to measurement uncertainties, this mapping would be noisy and not necessarily an accurate representation of the local variation of properties. Besides, the problem of averaging the identified parameters to get a representative set of properties is not trivial. Because we are more interested in a set of properties representing the global response of the tissue, a global identification technique based on the total strain energy is proposed.

The strain energy density *w* stored in the membrane can be expressed as (Reddy, 2017):

(12) $$\begin{array}{r} w=\int_{0}^{E_{ij}}\sigma_{ij}dE_{ij} \end{array}$$

The total strain energy *W*_exp_ for the membrane is then obtained by integration over the whole domain:

(13) $$\begin{array}{r} W_{exp}=\int_{V}w\;dV \end{array}$$

Knowing each element stress, strain and volume, this quantity was calculated for each time frame based on numerical integration and summation over the elements.

On the other hand, assuming an incompressible Ogden strain energy function, this quantity can also be expressed as:

(14) $$\begin{array}{r} W_{o}=\int_{V}\frac{\mu }{\alpha }\left(\lambda_{1}^{\alpha }+\lambda_{2}^{\alpha }+\lambda_{1}^{-\alpha }\lambda_{2}^{-\alpha }-3\right)dV \end{array}$$

Knowing the volume and the principal stretches of each element (derived from the deformation gradient tensor), this expression has only two unknowns: μ and α. Consequently, these parameters were identified using a non-linear optimization procedure, such as:

(15) $$\begin{array}{r} \alpha ,\mu =\text{argmin}\underset{i}{\sum }∥W_{exp}^{i}-W_{o}^{i}\left(\mu ,\alpha \right){∥}^{2} \end{array}$$

where superscript *i* represents a data frame.

Numerically, this non-linear optimization was performed using the fitnlm function in Matlab.

Similarly, an incompressible NeoHookean strain energy function was also used to identify the *C*_1_ parameter:

(16) $$\begin{array}{r} W_{nh}=\int_{V}C_{1}\left(\lambda_{1}^{2}+\lambda_{2}^{2}+\lambda_{1}^{-2}\lambda_{2}^{-2}-3\right)dV \end{array}$$

### 2.4. SALS

Small angle light scattering (SALS) was performed on a single sample to determine whether a preferred orientation of collagen fibers exists in the DAM. Briefly, the SALS system consists of an unpolarized 5 mW HeNe laser (λ = 632.8 nm; JDSU, Newbury, UK), focusing lens (fl = 150 mm; Edmund Optics Ltd, York, UK), automated sample positioner, projection screen and a CMOS USB camera mounted on a linear guide rail (Gaul et al., 2017). The device passes a 150 μm diameter laser beam through the tissue specimen which scatters light according to the internal fiber architecture. The resulting scattered light distribution is analyzed using a custom Matlab code (MathWorks, Cambridge, UK) to determine orientation and alignment information about the specimen as detailed elsewhere (Gaul et al., 2017). Due to sample size limitations imposed by the SALS system, the sample was cut into 6 rectangular sections of 25 × 10 mm and each section was analyzed individually. Vectors were spaced every 1 mm with each vector corresponding to a region of 150 μm^2^.

### 2.5. Craniotomy Simulation

**Figure 7:**
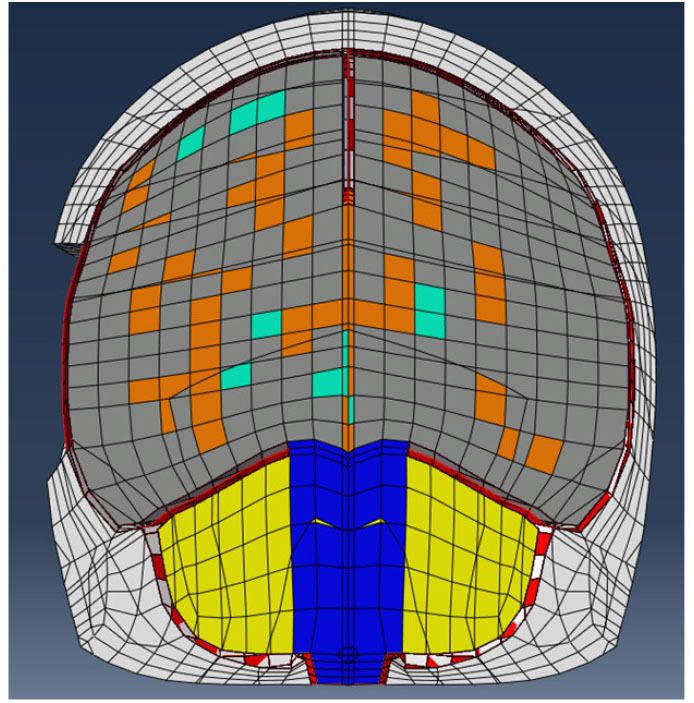
UCDBTM (Horgan and Gilchrist, 2003, 2004) with bone flap removed to simulate craniotomy with skull section removed to simulate a craniotomy procedure. Various colored elements represent different biological tissues (white = bone, gray = gray matter, orange = white matter, green = ventricles, yellow = cerebellum, blue = brain stem, red = membranes).

Following the mechanical evaluation of the DAM and the determination of an appropriate strain energy density function (Ogden model), finite element simulations were undertaken to highlight the potential applications of this study. Using Abaqus 6.14 software, the UCDBTM (Horgan and Gilchrist, 2003, 2004) (an existing finite element head trauma model developed at University College Dublin) was used to simulate a simplified craniotomy procedure. The mechanical properties of the biological tissues are given in Table 1. A section of skull measuring ~46 × 48 mm was removed from the UCDBTM as shown in Figure 7. Here, brain swelling, as experienced following TBI, was simulated using a thermal expansion method, where the coefficient of thermal expansion was 0.02°C^−1^ with an applied temperature increase of 1°C. In an unconstrained environment, this will generate thermal strains ε=0.02, which corresponds to a volumetric brain swelling of 6%. When a structure is not free to expand, such as the brain in the skull cavity, the change in temperature will cause stress. In this case, the true volumetric expansion will be dictated by the stiffness of the enclosing structures, in this case the DAM in the opened area. While this approach does not represent the physiological swelling process (Lang et al., 2014), the thermal expansion approach has been previously adopted by (Weickenmeier et al., 2017) for the simulation of brain swelling in craniotomies. The response of the UCDBTM using both the original meninges material properties, as described in (Horgan and Gilchrist, 2004), and the present material properties evaluated in inflation tests (detailed in Table 2), are contrasted with respect to the maximum principle strain in the brain and the maximum protrusion of the brain outside the bone flap. As high deformations were expected, geometric non-linearities were accounted for (NLGEOM option in Abaqus), so that the only difference between the two simulations was the material model of the DAM.

**Table 1:** Mechanical properties of UCDBTM (Horgan and Gilchrist, 2003, 2004).

**Tissue**	**Model**	**Density [kg/m3]**	**Poisson's ratio**	**Model parameters**	**# Elements**
Cerebellum	Viscoelastic	1,060		*G*_0_= 10 kPa *G*_∞_= 2 kPa Decay constant = 80^−*s*^
K = 2.19 GPa	780
Gray matter	Viscoelastic	1,060		*G*_0_= 10 kPa *G*_∞_= 2 kPa Decay constant = 80^−*s*^ K = 2.19 GPa	5,004
Brain stem	Viscoelastic	1,060		*G*_0_= 22.5 kPa *G*_∞_= 4.5 kPa Decay constant = 80^−*s*^ K = 2.19 GPa	220
White Mater	Viscoelastic	1,060	0.499	*G*_0_=12.5 kPa *G*_∞_= 2.5 kPa Decay constant = 80^−*s*^ K = 2.19 GPa	1,105
Cortical bone	Linear elastic	2,000	0.22	E=15 GPa	4,160
Trabecular bone	Linear elastic	1,300	0.24	E = 1 GPa	4,096
Pia mater	Linear elastic	1,130	0.45	E = 11.5 MPa	2,786
CSF	Linear elastic	1,000	0.499	E = 0.15 MPa	2,874
Facial bone	Linear elastic	2,100	0.23	E = 5.5 GPa	406
DAM (Horgan and Gilchrist, 2003, 2004)	Linear elastic	1,130	0.45	E = 31.5 MPa	2,155
**DAM (current)**	**Ogden (*****n*** **=** **1)**	**1130**	**0.49**	**μ** **=** **0.23 MPa**, **α** **=** **8.2**	**2,155**

*Current mechanical properties of the DM are provided in boldface*.

**Table 2:** DAM properties identified with the inflation test for the 10 samples (two for each pig, left side and right side).

				**Ogden**			**Neo-Hookean**		
**Pig**	**Side**	**Thickness**	**Burst pressure**	**μ**	**α**	**R^2^**	***C*_1_**	**R^**2**^**	**Rupture stress**
		**[μm]**	**[kPa]**	**[kPa]**	**[-]**	**[-]**	**[kPa]**	**[-]**	**[MPa]**
1	L	360	193	11.9	13.8	0.983	345	0.718	^*^
	R	359	206	481	5.39	0.997	905	0.972	2.00
2	L	377	127	177	8.61	0.988	598	0.942	^*^
	R	381	183	497	4.50	0.987	654	0.970	2.52
3	L	361	^†^	^†^	^†^	^†^	^†^	^†^	^†^
	R	390	141	273	5.64	0.997	490	0.973	^*^
4	L	400	150	136	10.3	0.990	591	0.943	1.72
	R	327	^†^	^†^	^†^	^†^	^†^	^†^	^†^
5	L	405	150	59.6	9.12	0.992	295	0.869	^*^
	R	394	^†^	^†^	^†^	^†^	^†^	^†^	^†^
Mean		375	164	234	8.19	0.991	554	0.912	2.08
St. Dev.		24	30	193	3.29	0.005	205	0.093	0.41

* *Rupture not in area of interest*.

† *Experiment failed*.

## 3. Results

### 3.1. Tensile Testing

The measured thickness of the DAM using Venier calipers was 400 ± 100 μm. For each uniaxial tensile test performed, a stress-strain curve was calculated. From these curves, the average failure strain was found to be 22 ± 4%, and the average ultimate tensile strength was 6.91 ± 2.00 MPa. Each curve was fit to both the Ogden and the NeoHookean material models using the lsqnonlin function in MATLAB.

**Figure 8:**
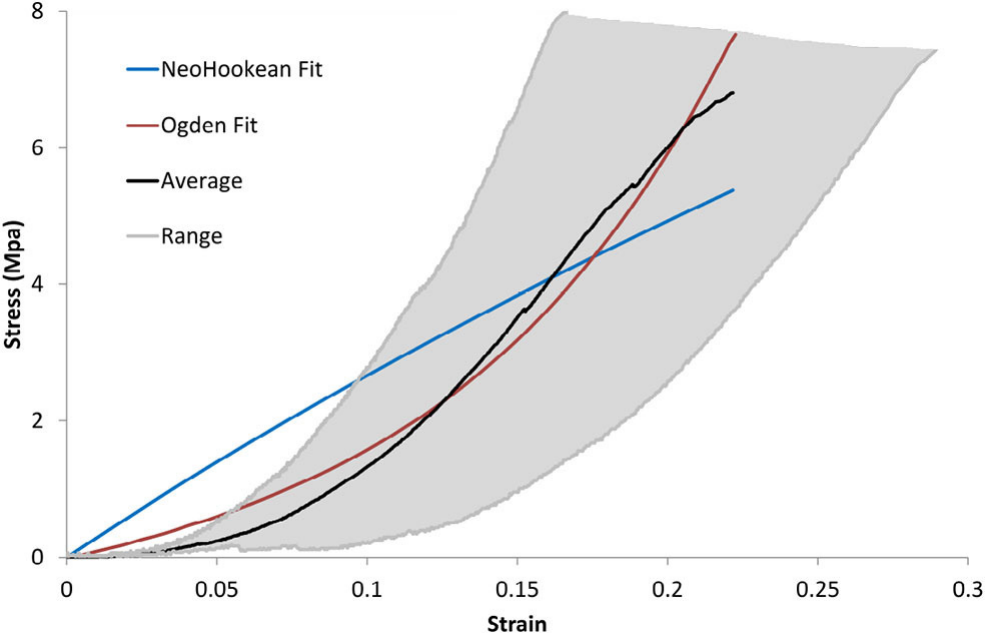
Average and range of stress-strain relationships of DM for uniaxial tension tests and representative best fit Ogden model.

It can be seen from Figure 8 that the Ogden model captures the non-linear response of the DAM well, with an average *R*^2^=0.98, while the NeoHookean model fails to capture the toe region of the response with an average *R*^2^=0.82. The average Ogden material parameters for all samples tested was μ = 450 ± 190 kPa and α = 16.55 ± 3.16. As per Equations (5) and (6) this results in a shear modulus, μ_*s*_ of 3.55 ± 1.46 MPa and a Young's modulus, *E*, of 10.64 ± 4.39 MPa. The average NeoHookean material parameter for all samples tested was *C*_1_ = 5.87 ± 2.90 MPa, which corresponds to a shear modulus, μ_*s*_ of 11.74 ± 5.79 MPa. Figure 8 shows the average stress-strain relationship and the entire range of the samples tested in uniaxial tension. Also shown is a representative fit of the Ogden model and the NeoHookean model to the average stress-strain curve.

**Figure 9:**
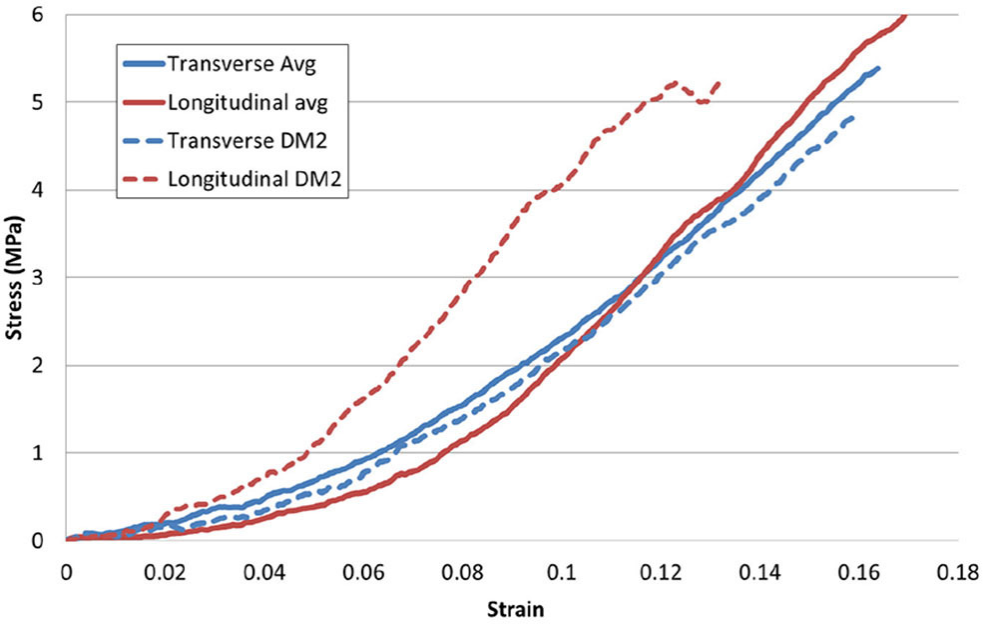
Directional effects of the DM. The average response of samples (shown as solid lines) in orthogonal directions is not statistically different, however, longitudinal samples appear somewhat stiffer, especially when inter-subject variability is eliminated, e.g., as shown by dashed lines representing the longitudinal and transverse response from a single DM.

Analysing average stress strain curves in both the longitudinal (*N* = 17) and the transverse (*N* = 8) orientations, no clear difference was evident (see Figure 9). However, when inter-subject variability was removed by comparing samples only from the same DAM, it was observed that longitudinal samples appeared stiffer than transverse samples also shown, for example, in Figure 9. This was a trend observed for all three DAMs tested in this manner. However, a two tail *T*-test revealed that the Ogden model parameters for samples in the longitudinal and transverse direction were not statistically different (*p* = 0.13 for μ, *p* = 0.87 for α). This indicates that while there is some anisotropy evident, this is not statistically significant within the current results and the global material behavior of the cerebral DAM can be considered isotropic. This will be discussed further in section 3.3.

### 3.2. Inflation Testing

**Figure 10:**
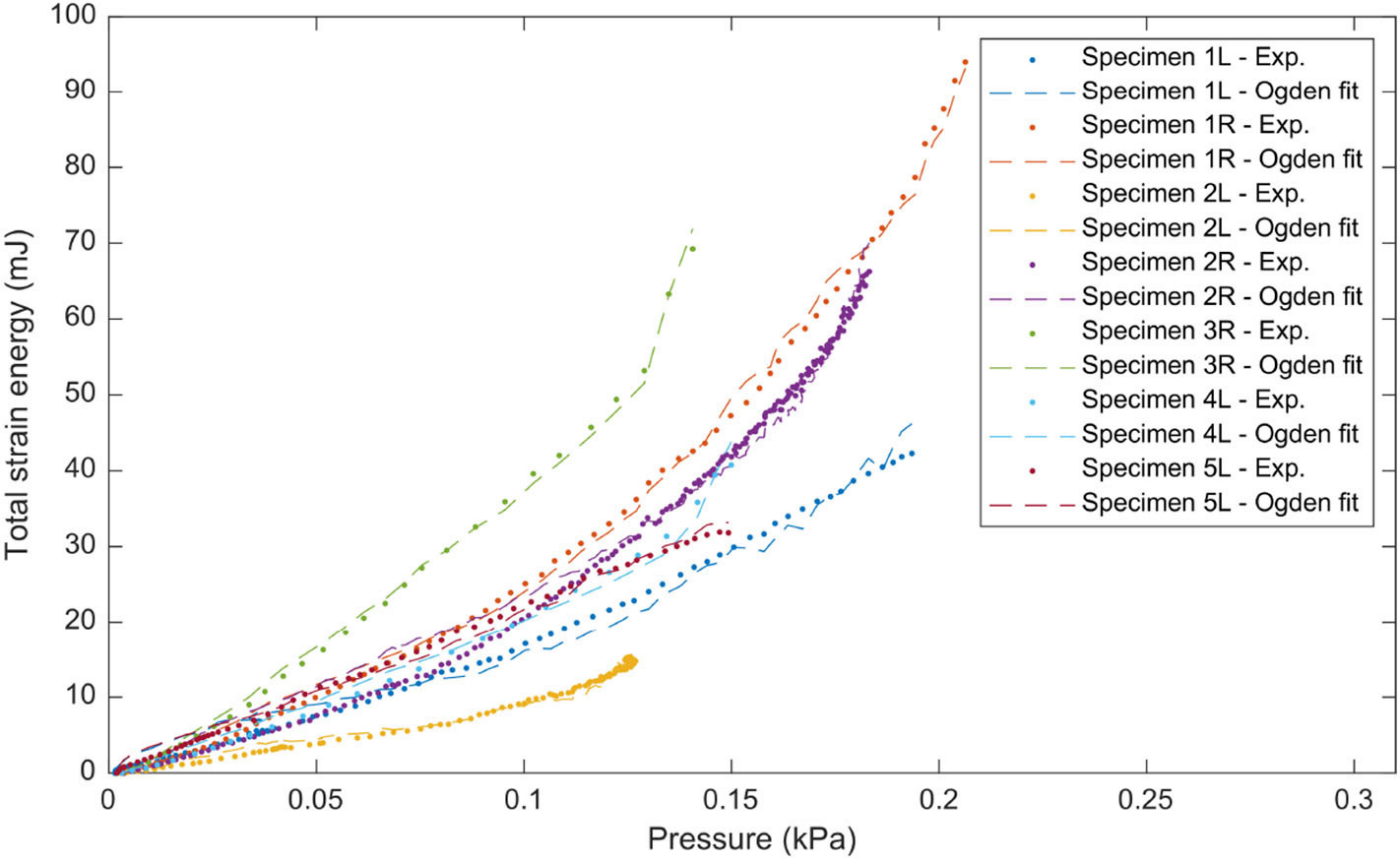
Total strain energy vs. pressure for all DM samples of the inflation tests. Experimental values are shown as dots and the fit with the Ogden strain energy function in dashed lines.

The results of the inflation testing are presented in Table 2. An example of the sDIC reconstruction is shown in Figure 5, and an example of a successful burst is shown in Figure 6. The mean measured thickness for all specimens using the laser triangulation method was 375 ± 24 μm. From the 10 samples, sDIC reconstruction failed for 3 due to the speckle pattern being affected by water droplets. The mean constitutive parameters identified for the Ogden model were μ=234±193 kPa and α=8.19±3.29. The Ogden model fit the experimental data well with a mean coefficient of determination of *R*^2^=0.99, as can be seen in Figure 10. Also, the mean confidence intervals were ±20 kPa for μ and ±0.38 for α with confidence level of 95% as returned by the non-linear optimization. This shows that our identification procedure is sensitive to both parameters of the model. The mean identified parameter for the NeoHookean model was *C*_1_=554±205 kPa. However, as this model has a more linear response, it fit the data relatively poorly with a mean *R*^2^ of 0.91. Rupture data was successfully obtained in the area of interest for three specimens only. The mean value of rupture stress was 2.08±0.41 MPa.

### 3.3. Orientation

**Figure 11:**
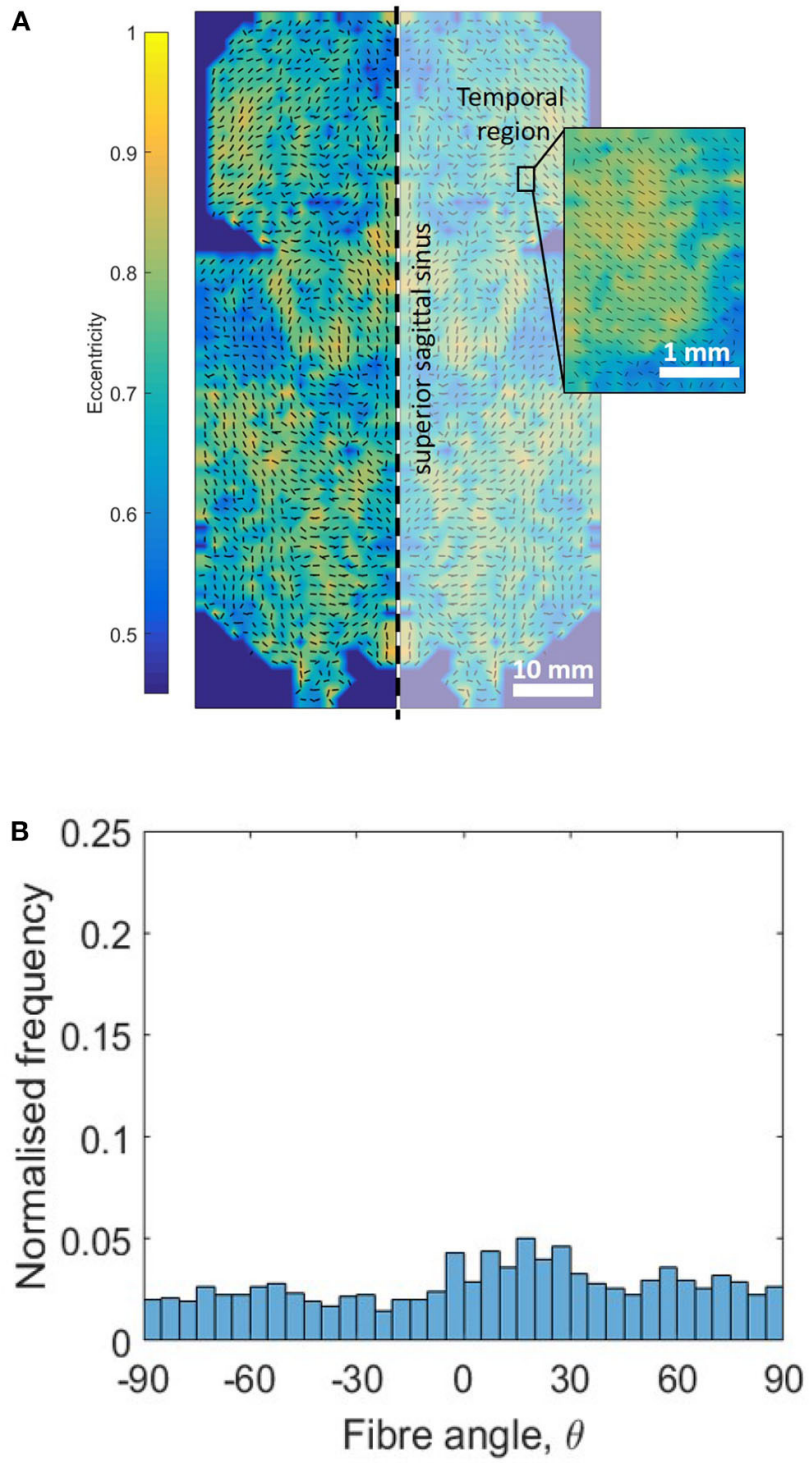
Evaluation of DAM microstructure. **(A)** Global and local (inset) SALS contour plots of fiber orientation and alignment, where eccentricity values (0–1) represent the level of fiber alignment. **(B)** Orientation distribution plot of global DM fiber directions. Only the left half of the DM was analyzed, the right half was generated through mirror symmetry.

SALS contour plots were generated for one half of the DAM and the results were mirrored along the superior sagittal sinus for illustration (Figure 11A). The fiber alignment is defined by eccentricity (values of 0–1) where areas of high eccentricity (> 0.7) have fibers highly aligned with the vector directions indicated and areas with low eccentricity (<0.7) have a low degree of fiber alignment, or highly dispersed fibers. Results indicate that there are local regions of high alignment (as shown in yellow and inset), however there is no global preferred orientation, as seen in Figure 11B. The lack of a dominant angle in Figure 11B indicates that the DAM is globally isotropic. This result is also corroborated by the statistical analysis presented in section 3.1, which found no statistical difference between the Ogden model parameters for samples in the longitudinal and transverse direction.

### 3.4. Craniotomy Simulation

**Figure 12:**
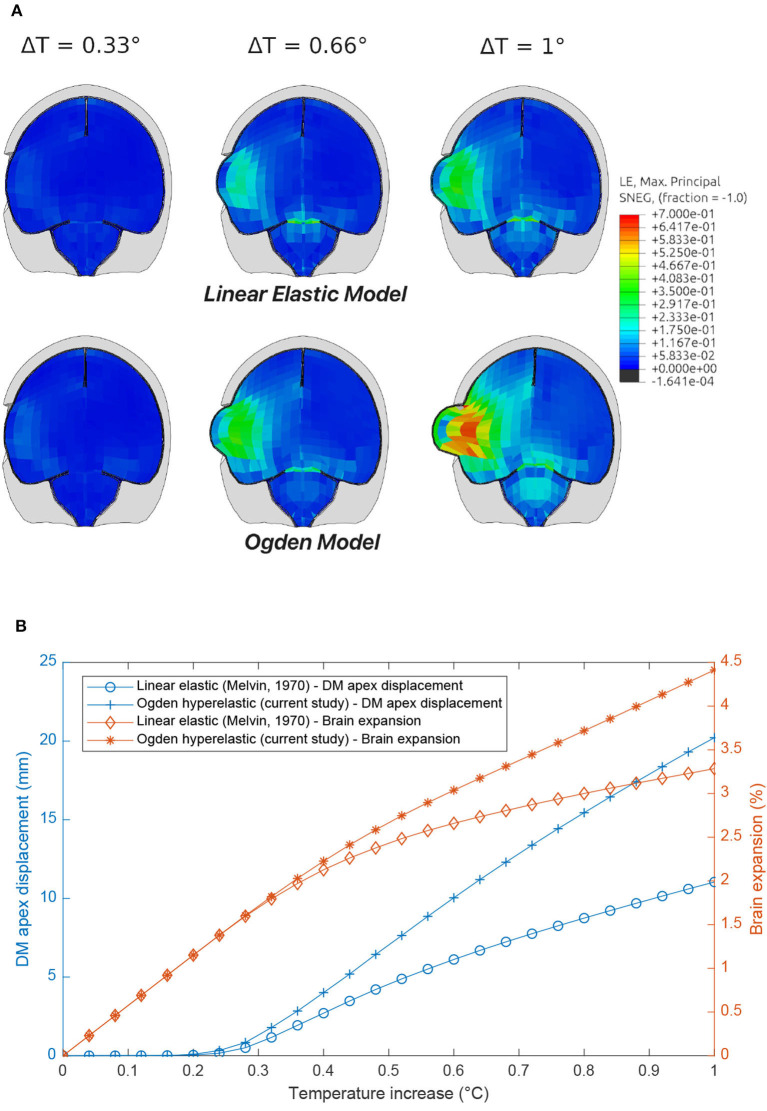
Comparison of craniotomy simulation using the Linear Elastic Model (Horgan and Gilchrist, 2004) and Ogden Model (present study) induced by brain swelling simulated by thermal expansion [1–2% swelling corresponds to typical volumetric expansion seen at high altitudes (Morocz et al., 2001)]. **(A)** Maximum Principal Strain contour plots. **(B)** DM apex displacement and brain expansion vs. temperature increase.

It can be seen in Figure 12 that the linear elastic and Ogden material models predict two significantly different responses, with the stiffer linear elastic model predicting lower Maximum Principal Strains and a less pronounced protrusion of the brain following the craniotomy, caused by a 26% lower brain expansion. Stress fields were also highly affected by the DAM properties: brain (intracranial) pressure ranged from 13–15 to 46–50 kPa for the linear elasic and Ogden models, respectively. This emphasizes the fact that the material properties of the DAM have a significant impact on the overall response of the brain.

## 4. Discussion

Comparing the main results under uniaxial testing of the current study to previous authors (see Table 3), we find that our failure strain of 22 ± 4% falls between the values reported by Van Noort et al. (1981a) and McGarvey et al. (1984) of 18.72 and 31.7%, respectively and that our Ultimate Tensile Strength of 6.91 ± 2.00 MPa also falls between the values reported by Van Noort et al. (1981a); McGarvey et al. (1984) of 4.7 and 9.41 MPa, respectively. The determined average material parameters were μ = 450 ± 190 kPa and α = 16.55 ± 3.16 for the Ogden model and *C*_1_ = 5.87 ± 2.90 MPa for the NeoHookean model. Based on the calculated average coefficient of determination (*R*^2^=0.98 for Ogden model and *R*^2^=0.82 for NeoHookean), it has been determined that the Ogden model offers a better fit to experimental data. Two previous authors, Maikos et al. (2008) and De Kegal et al. (2017), have also suggested that the Ogden model offers the best fit to experimental data and that it captures the non-linear region of the stress-strain response well. Again, the reported material parameters in the current study lie between the values reported by Maikos et al. (2008) (μ = 420 kPa) and De Kegal et al. (2017) (μ = 660 kPa). Given the close comparison between these results, porcine DAM can be considered a good model for human DAM as previously suggested by Frink et al. (2011) and Mazgajczyk et al. (2012).

**Table 3:** Summary of results found in previous tensile investigations.

**Summary of tensile testing**				
**References**	**Species**	**Thickness**	**Model parameters**	**Descriptive values**
Galford and McElhaney (1970)	Human	–	MK: E_*s*_ = 29.65 MPa, E_*l*_ = 3.38 MPa	–
Van Noort et al. (1981a)	Human	–	–	UTS = 4.7 MPa, FS = 18.4%
McGarvey et al. (1984)	Human	–	–	UTS = 9.41MPa ± 1.54, FS = 31.7% ± 1.6
Bylski et al. (1986)	Fetal human	0.57 mm	MR: C = 1.18 MPa, α = 0.25	–
Maikos et al. (2008)	Rat	0.8 mm	OG: μ = 0.42 MPa ± 0.19, β = 32.9 ± 6.65	UTS = 2.49 MPa ± 2.03, FS = 39% ± 13
De Kegal et al. (2017)	Human	1.06 mm ± 0.22	OG: μ = 0.66 MPa ± 0.38, β = −1.09 ± 3.19	–
MacManus et al. (2017a)	Porcine	–	NH and QLV; μ = 0.019 MPa ± 0.0085	–
Present study	Porcine	0.4 mm ± 0.1	OG: μ = 0.45 MPa ± 0.19, α = 16.55 ± 3.16	UTS = 6.91 MPa ± 2.00, FS = 22% ± 4

*MK, Maxwell-Kelvin; MR, Mooney Rivlin; OG, Ogden; NH, NeoHookean; QLV, Quasi Linear Viscoleasticity; E_s_, Storage Modulus; E_l_, Loss Modulus; UTS, Ultimate Tensile Strength; FS, Failure Strain*.

The results of the bulge inflation testing confirmed that the Ogden model is a suitable choice of constitutive model since the mean coefficient of determination here was *R*^2^=0.99. Inflation results, however, differ somewhat from the results of the uniaxial testing, highlighting that the identification of constitutive parameters is dependent on the test method. Similar observations have been made by Lari et al. (2012) for sclera, where they compared the response of uniaxial tension and inflation experiments suggesting that testing conditions in the uniaxial tests can induce artifacts due to sensitivity to tissue gripping techniques, sample cutting, and Saint-Venant related issues. Regarding the current experiments, the identified Ogden material parameters were significantly less for bulge inflation testing than unaxial tensile testing (52% less for μ and 49% less for α). Similarly, bulge inflation testing revealed a rupture stress of 2.08±0.41 MPa, significantly below the Ultimate Tensile Strength of 6.91 ± 2.00 MPa. While rupture data is based only on the analysis of three specimens, it is included here given that no other studies have previously determined the rupture strength of the DAM through bulge inflation testing. Interestingly, it can be seen in Figure 6 that tissue failure is not located in the same region as peak stress, which was observed for all three specimens. Although surprising, this observation has also been reported for arterial tissue (Cavinato et al., 2019), suggesting that rupture mechanisms may be better explained by an understanding of the microstructure and composition heterogeneities in the tissue. As DAM is a highly fibrous medium, local rearrangement of the microstructure depending on the loading conditions may also explain the discrepancy between uniaxial and biaxial (inflation) identified parameters. It should be noted that the test specimens came from 22 week - 23 week old pigs. It is well-known that the material properties of soft tissues are affected by age (Thibault and Margulies, 1998; MacManus et al., 2017b) and the results presented here may not be generalizable across all ages.

The average thickness of samples in the current study measured by Vernier calipers was 0.4 ± 0.1 mm and for those determined through the laser triangulation method, this was 375 ± 24 μm, indicating broad agreement between the two methods. The mean figure is lower than that of De Kegal et al. (2017) and Galford and McElhaney (1970) who reported values of 1 mm for human DM. Bylski et al. (1986) however reported an average thickness of 0.57 mm for fetal human DM and suggested that the thickness of the DM increases with age. Furthermore, Galford and McElhaney (1970) compared the thickness of human and monkey DM and found that human DM was thicker, while Maikos et al. (2008) reported a DM thickness of 0.8 mm for rats. Given that samples used in the current study are from adolescent pigs, this may explain somewhat the lower thickness values obtained when compared to older human samples. This difference may also be attributed, in part, to the different measurement techniques employed. O'Leary et al. (2013) noted that optical measurement techniques, such as those employed by De Kegal et al. (2017), can overestimate the thickness of soft tissues by an average of 23%, while Vernier calipers can underestimate the thickness by an average of 8%.

Tensile tests were carried out at a speed of 50 mm/min, corresponding to a strain rate of ~0.01 s^−1^ and comparable to the strain rate applied in De Kegal et al. (2017). De Kegal et al. (2017) found no evidence of hysteresis and therefore concluded that the DM could be considered purely elastic at lower strain rates. Conversely, other authors have observed substantial viscoelastic behavior and modeled the meninges as either quasi-linear viscoelastic or linearly viscoelastic (Galford and McElhaney, 1970; MacManus et al., 2017a). The present study, however, did not consider the effects of viscoelasticity and therefore care should be taken when comparing these results to testing carried out at dynamic rates (Maikos et al., 2008; MacManus et al., 2017a).

**Figure 13:**
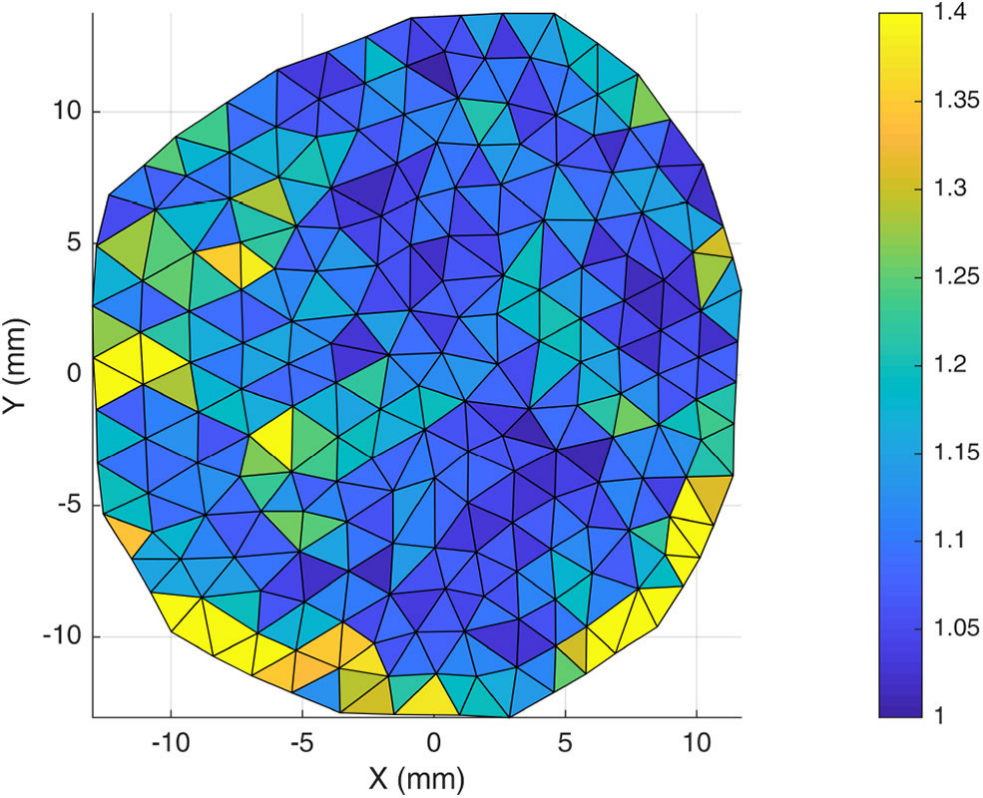
Stretch ratio, calculated as first principal stretch/second principal stretch, for a given DAM. For an isotropic material this should be 1. It can be seen that the stretch ratio is close to 1 in the centre and only deviates from 1 at the edge.

Previous authors have all concluded that the behavior of the DM is globally isotropic (Van Noort et al., 1981a; Hamann et al., 1998; De Kegal et al., 2017). This statement however, is usually qualified with a suggestion that there is local anisotropy and regions of aligned collagen fibers. For this reason, tensile testing and SALS was conducted with a view to determining whether the meninges really should be considered as an isotropic material. SALS orientation analysis shows global isotropy in the DAM, highlighted by the lack of a dominant direction in the orientation distribution plot shown in Figure 11. There are, however, regions of local fiber alignment, such as the temporal region which has been reported elsewhere for human DM (Hamann et al., 1998). Closer analysis of our inflation tests in Figure 13 demonstrates that the ratio of the first principal stress/the second principal stress is close to one and only deviates from one at the edge. Given that the inflation tests involve an equibiaxial tension state, this is further evidence of the globally isotropic response of the DAM. Furthermore, tensile tests revealed that there was no statistically significant difference between samples tested in the longitudinal and transversal directions. When samples from the same DAM only were compared (thereby eliminating inter-subject variability), the longitudinal samples appeared to be slightly stiffer. This difference, however, was not statistically significant within the test population. To definitively determine whether the cerebral meninges is isotropic, a much larger sample size would be required. However, as previous authors have concluded, and in the absence of statistically significant data to the contrary, the cerebral meninges can be considered isotropic.

The analysis of the inflation tests were performed under the assumption that the DAM was isotropic and incompressible. By identifying a single set of parameters for each sample, it was also assumed that the thickness and material properties were homogeneous throughout the membrane. Hence, homogeneous stresses and strains were expected. From Figure 6 it can be seen that the stress was not always homogeneous; this may be due to regions of local fiber alignment, non-uniform thickness across the sample, or heterogeneities in the tissue constituents. The thickness measurement device allows the mapping of local thickness, and its direct use should be considered in future works. On the other hand, Chauvet et al. (2010) performed an analysis on the displacement and strain field in uniaxial tension showing that the displacement field appeared homogeneous along the sample. It was therefore concluded that the meninges could be considered homogeneous. The compressibility of the meninges has not been extensively discussed in the literature, but given that the meninges is primarily composed of water it is common to assume that it is incompressible.

Bulge inflation testing coupled with sDIC and thickness measurement was found to offer definite advantages in terms of reliability of the results, although the experimental setup and subsequent analysis is more involved. Firstly, it provides direct access to stresses without prior knowledge of the material constitutive behavior. Secondly, direct measurement of local displacement on the surface of the specimen avoids the need for arbitrary assumptions at the clamped edges (e.g., no slippage), which is often problematic to verify. Finally, it is important to note that bulge inflation testing, compared with uniaxial tensile testing, offers a more biofidelic representation of the *in-vivo* loading conditions experienced by the DAM which is under intracranial pressure. Given that the evaluated material parameters vary depending on the test method, it is suggested that future finite element simulations employ material parameters evaluated through bulge inflation testing.

The purpose of simulating the craniotomy procedure was to emphasize the important role of the meninges in predicting the response of the human head under various loading conditions. A further objective was to highlight that even under physiological strains (e.g., such as those experienced by someone following TBI), a non-linear description of the meninges is important. Indeed, a recent publication by Li et al. (2017) has highlighted that non-linear models of tissues should replace linear models in head models for more realistic simulation outcomes.

Finally, while ideally tissue should be tested in its physiological state, or as close as is possible, the tissue here has been tested between 4 and 24 h post-mortem at ambient conditions. There is no global agreement on the effect of prolonged storage of brain tissue on mechanical properties, with some authors concluding there is minimal effect up until 5 days post-mortem (Budday et al., 2015), while others conclude that as short as 6 h of storage can affect the properties (Garo et al., 2007). As concluded by Budday et al. (2019) in their review paper of brain tissue, we have considered that, provided the tissue is kept sufficiently hydrated, the degeneration process of post-mortem tissues is negligible. Similarly, it has been previously reported that increasing the temperature from ambient conditions to physiological conditions (37°C) significantly reduces the stiffness of brain tissue (Pogoda et al., 2014). Contrary to this, a number of authors have also reported the opposite; that increasing the temperature from ambient to 37°C has negligible effect (Rashid et al., 2012). As stated above, best practice is to test as close as possible to physiological conditions, and the fact that samples were tested at ambient conditions may have an effect on the present results.

## 5. Conclusion

It was confirmed that the global behavior of the porcine DAM can be considered isotropic (although it does contain local regions of highly aligned collagen fibers). The mechanical behavior of the DAM is well-described by the non-linear Ogden hyperelastic model (average parameters determined through bulge inflation testing); μ=234±193 kPa and α=8.19±3.29, which better captures the non-linear toe region in the stress-strain response compared with existing linear elastic models. A finite element simulation of a craniotomy procedure revealed that the mechanical behavior of the meninges has a large effect on the overall response of the brain and that existing FE head models should be updated to include a non-linear description of the meninges.

## Data Availability Statement

The datasets generated for this study are available on request to the corresponding author.

## Ethics Statement

Ethical review and approval was not required for the animal study because only post-mortem animal tissue obtained from a local slaughterhouse was used for this study. The study is therefore exempt from ethical approval.

## Author Contributions

AN and BP contributed to the conception, design and oversight of the project, and wrote the first draft of the manuscript. MG and CL contributed to the design of the project and revised the work critically. LC, FM, DM, and RG completed the experimental aspects of the work and wrote the corresponding sections of the manuscript. All authors contributed to the manuscript revision.

## Conflict of Interest

The authors declare that the research was conducted in the absence of any commercial or financial relationships that could be construed as a potential conflict of interest.

## Footnotes

^1^Following the convention used in MacManus et al. (2017a), the current samples are referred to as *dura-arachnoid mater* (DAM), accurately reflecting the samples' composition. When referring to previous works, they are described as per the author's description i.e., *dura mater* or meninges.
